# It Takes Two to Tango: Synergistic Expandable Graphite–Phosphorus Flame Retardant Combinations in Polyurethane Foams

**DOI:** 10.3390/polym14132562

**Published:** 2022-06-23

**Authors:** Yin Yam Chan, Bernhard Schartel

**Affiliations:** Bundesanstalt für Materialforschung und-prüfung (BAM), Unter den Eichen 87, 12205 Berlin, Germany; yin-yam.chan@bam.de

**Keywords:** synergy, phosphorus-containing flame retardant, expandable graphite, polyurethane foams

## Abstract

Due to the high flammability and smoke toxicity of polyurethane foams (PUFs) during burning, distinct efficient combinations of flame retardants are demanded to improve the fire safety of PUFs in practical applications. This feature article focuses on one of the most impressive halogen-free combinations in PUFs: expandable graphite (EG) and phosphorus-based flame retardants (P-FRs). The synergistic effect of EG and P-FRs mainly superimposes the two modes of action, charring and maintaining a thermally insulating residue morphology, to bring effective flame retardancy to PUFs. Specific interactions between EG and P-FRs, including the agglutination of the fire residue consisting of expanded-graphite worms, yields an outstanding synergistic effect, making this approach the latest champion to fulfill the demanding requirements for flame-retarded PUFs. Current and future topics such as the increasing use of renewable feedstock are also discussed in this article.

## 1. Introduction

As fire safety has always been a major concern, fire protection is in high demand. One of the key approaches to improving fire protection entails adding flame retardants to polymeric materials, because most synthetic polymers are easily ignited due to their high content of hydrocarbons, an excellent fuel for fires. Currently, efficient flame retardancy is achieved through specific solutions tailored to different kinds of polymeric materials, as they have different properties [1,2,3]. Flame retardancy is specific with respect to the flame-retardant mechanisms and to the flame retardant’s reactions with polymeric materials [4,5,6,7] and with other ingredients, such as additional flame retardants, fillers/fibers, additives, adjuvants, and synergists [8].

Flame retardancy is specific with respect to the protection goal and fire scenario; ignition scenarios require different approaches from developing fires or fully developed fires [9]. Different flame retardants are even favored for the same polymer depending on whether it is applied in bulk, as a composite, or in the form of fibers or foam. Thus, a multitude of different flame retardants in various combinations are used to protect the entire spectrum of foam-containing consumer goods [10]. Generalized approaches of efficient combinations working in different matrices, such as flame retardants containing Br combined with Sb_2_O_3_ or ammonium polyphosphate (APP) with pentaerythritol (PER), are rare; furthermore, environmental concerns mean that halogen-free systems are preferred. One of the champions among the currently proposed flame retardants that has found application in today’s products is the synergistic combination of expandable graphite (EG) with a phosphorous flame retardant (P-FR). This approach has become legendary for the excellent flame retardancy it provides to polyurethane foams (PUFs) [11,12,13,14]. In this feature article, we turn the scientific spotlight on the concept, mechanisms, and role of the synergistic combinations of EG and P-FRs in PUFs in order to evoke the applause this approach deserves [15,16,17,18]. 

### 1.1. Polyurethane Foams (Flexible and Rigid)

Polyurethane foams (PUFs) have been used in a wide range of applications because their physical and mechanical properties can be customized by changing the chemical composition. PUFs are divided into two main categories: flexible polyurethane foams (FPUFs) and rigid polyurethane foams (RPUFs) [19,20,21]. The main chemicals used in the formulation for PUFs are polyols, isocyanates, catalysts, surfactants, and blowing agents. The differences in the physical and mechanical properties of FPUFs and RPUFs depend mainly on the chemical characteristics of the reactants—the polyols and the isocyanates. Urethane linkages form in PUFs through a polyaddition reaction between the hydroxyl group of polyols and the NCO groups of isocyanates [22,23,24]. Nevertheless, the functionality of polyols and the type of isocyanate used are different in RPUFs and FPUFs. The relationship among the average molecular weight, functionality, and OH value of polyols is shown in Equation (1). These parameters are key characteristics that define the properties of polyols and ultimately affect the property profile of polyurethanes when polyols react with diisocyanates. For example, increasing the OH value leads to a higher crosslink density in polyurethane.

(1) $$M_{n}=\frac{z\times 56106}{OH\;value}$$

where *M_n_* and *z* are the average molecular weight and the functionality, respectively.

For RPUFs, polyols with shorter chains exhibiting higher functionality (*z* = 2.5–5) are combined with polymeric diphenylmethane diisocyanate (pMDI) in order to generate more crosslinks, providing more strength, thereby increasing rigidity. As a result, the apparent density of RPUFs is higher than that of FPUFs due to the former’s higher crosslink density [25,26]. Accordingly, RPUFs are more commonly used in construction, transportation, and refrigeration because of their extremely low thermal conductivity due to their closed-cell structure, which is superior to that of other commercially available insulation materials [27,28]. Because FPUFs are so flexible, long-chained polyols with lower functionality (*z* = 2–3) and toluene diisocyanate (TDI) are typically the main components added [29]. The open-cell structure of FPUFs contains cell window, strut, and strut join. Such morphology offers different degrees of cushioning, making it a frequent material choice in furnishings, automotive seating, mattresses, and packaging. 

### 1.2. Polyisocyanurate Foams and Polyurethane Foams

Although polyisocyanurate foams (PIRFs) and polyurethane foams (PUFs) have similar chemical compositions, PIRFs exhibit considerably better flame retardancy than PUFs. As PUFs have a more balanced equivalent weight ratio of the isocyanate group and the hydroxyl group of polyols (NCO/OH ratio ≈ 1.05–1.1), they mainly form urethane linkages, while the much higher excess amount of isocyanates in PIRFs usually yields isocyanurates via trimerization reaction. Due to the higher content of ring structures, PIRFs produce more char during burning, which ensures superior fire behavior. Günther et al. [27] compared the morphology of PIRF and RPUF residues with cone calorimeter measurements; the better fire behavior of PIRFs was attributed to the dense, thick cellular structure residue retained to some degree as thermal insulation, while RPUFs showed a thin, brittle residue layer. Therefore, the residue from PIRFs protects the underlying material better than the residue from PUFs during burning.

### 1.3. Flammability and Smoke Toxicity during Burning of Polyurethane Foams

**Figure 1:**
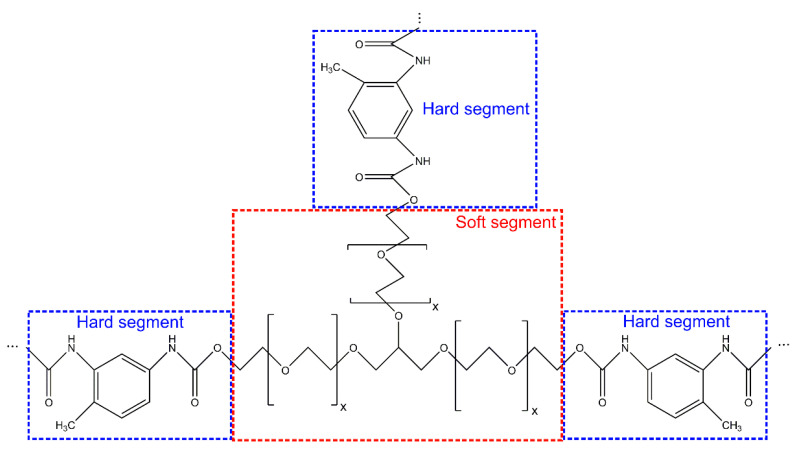
Chemical structure of flexible polyurethane foams.

Despite all the advantages enjoyed by PUFs, one of their major problems is high flammability. Regardless of whether RPUFs or FPUFs are used, both forms of PUFs present highly porous and cellular-structured material that easily catches fire. Because the cell walls and struts are thermally thin, they can be heated to the ignition temperature swiftly, causing early ignition [30]. Meanwhile, the low thermal conductivity makes the entire specimen thermally thick, which concentrates the heat at the surface, resulting in rapid flame spread. Therefore, the flame retardancy of polyurethane foams needs to be improved to meet high fire protection standards. Due to the open-cell structure and higher surface-to-mass ratio, FPUFs have higher flammability than RPUFs [31]. FPUFs show a lower tendency to char and a higher tendency to yield liquid products and thus collapse and yield pool fires. Figure 1, which illustrates the chemical structure, shows that PUFs are predominantly composed of combustible elements such as carbon and hydrogen, which increase the growth rate of fire. Apart from their flammability, PUFs evolve poisonous gases during burning, including carbon monoxide, nitrogen oxides, and hydrogen cyanide, as well as a large amount of smoke particles, all of which threaten human lives. The quality and quantity of poisonous gas evolved from the same material vary with different oxygen concentrations and burning temperatures [32,33].

### 1.4. Commercial Flame Retardants for PUFs

To cope with the flammability of PUFs, additive and reactive flame retardants are often introduced into the matrix to delay ignition and reduce heat release in the event of a fire, thereby slowing flame spread. Compared with RPUFs, it is more difficult to enhance structural frame retardancy of FPUFs. This is because flame retardants added physically often increase the viscosity of the polymer system and limit foam growth. The flame retardant is mainly embedded in the thin cell struts, causing the structure to collapse under the weight of the additive during the foaming process.

In the past, dispersing halogenated flame retardants such as organochlorine and organobromine compounds in polyurethane foams were very attractive for the industry, because they work effectively in the gas phase and greatly reduce heat release during burning [10]. However, the hydrogen halides released from halogenated flame retardants during burning are highly corrosive, are toxic to human beings, and may pollute the environment. Due to environmental and biological health concerns, some countries have already considered legislation restricting the use of flame retardants containing halogen. As a result, more and more halogen-free and environmentally friendly flame retardants have been developed and used in recent decades [34]. Today, dimethyl methylphosphonate (DMMP) [35], triaryl phosphates [36], melamine [31,37], aluminum hydroxide (ATH), expandable graphite (EG) [38,39,40], and ammonium polyphosphate (APP) [41,42] are common halogen-free additive flame retardants for polyurethane foams. Many studies have found that mixing two flame retardants or combining two or more flame-retardant elements in a single compound can increase the flame-retardant efficiency. This phenomenon is called synergism [43]. Li et al. [44] investigated the flame retardancy of RPUFs combined with DMMP and modified APP. They found that DMMP and modified APP enhanced flame retardancy through good coordination in the gas phase and the condensed phase. Wang et al. [45] synthesized a flame retardant containing phosphorus and nitrogen in RPUFs. The foam with the flame retardant formed a protective char layer, which enhanced flame retardancy. Tris(1-chloro-2-propyl) phosphate (TCPP) and tris(1,3-dichloro-2-propyl) phosphate (TDCP), both with two flame-retardant chemical elements (i.e., halogen and phosphorus), are still common additive flame retardants for polyurethane foams [10]. Most additive flame retardants deteriorate the morphology and mechanical properties of polymers. Hence, reactive flame retardants are an alternative to improve flame retardancy by chemically bonding to the polyurethane structure without excessively damaging the PUF structure. Phosphorous polyols are used as reactive flame retardants to replace petrochemical polyols in the formulation. Commercial non-halogenated phosphorous polyols such as Exolit^®^ OP 550 and Exolit^®^ OP 560 from Clariant AG (Muttenz, Switzerland) are successfully used in the industry. However, it is worth noting that FPUFs are sensitive to the hydroxyl number of polyols [46,47]. Higher hydroxyl values of polyols may cause the structure of FPUFs to collapse. Therefore, determining the appropriate amounts and types of polyols is the key to successful foaming.

## 2. Task

### 2.1. Burning Behavior of Rigid and Flexible Polyurethane Foams

**Figure 2:**
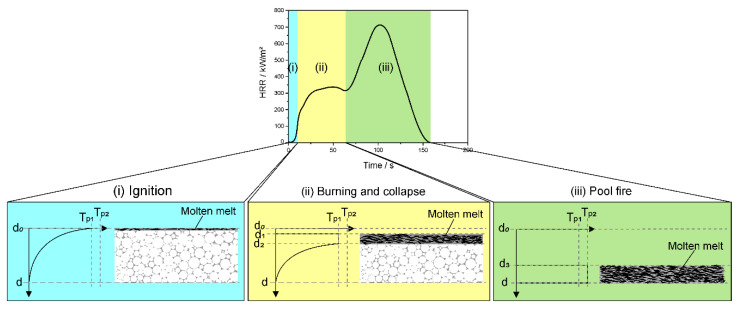
Temperature–thickness relationship of flexible polyurethane foams during different burning stages.

**Figure 3:**
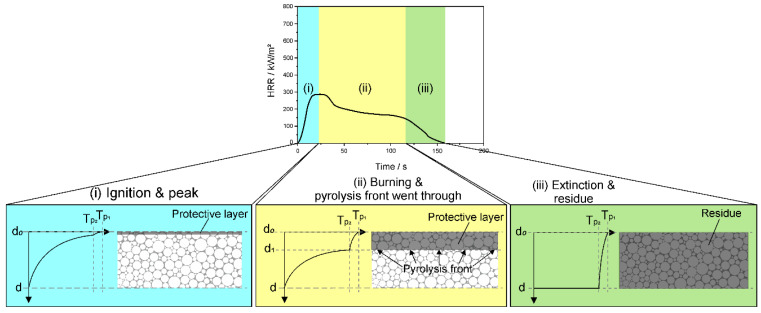
Temperature–thickness relationship of rigid polyurethane foams during different burning stages.

In terms of burning behavior, FPUFs can be ignited more easily than RPUFs, and fire propagates more quickly because of their lower density and open-cell structure [48,49], while RPUFs have a higher density and a closed-cell structure [26]. The curves of the heat release rate (HRR) of FPUFs from cone calorimeter measurements are displayed in Figure 2. For FPUFs, the curve exhibits three stages. In the first step (i), the surface of the foam is heated up; then, decomposition is initiated, and the foam ignites. According to the two-step decomposition of polyurethane, mainly, urethane bonds decompose, and the volatile pyrolysis products of the hard segments feed the flame. After this ignition stage (i), in stage (ii), the foam is covered by a molten layer of pyrolyzing polyurethane, such that the foam burns, collapses, and forms a pool of intermediate liquid pyrolysis products. After the first peak or plateau-like burning in stage (ii), the heat release rate surges to another, higher peak in stage (iii), because the remaining material burns in a violent pool fire [48,50,51]. The differences between the different burning stages can be described by the temperature–thickness relationship. Figure 2 and Figure 3 show the temperature–thickness relationship of FPUFs and RPUFs during different stages of burning, respectively. At the beginning of burning, individual cell walls or struts behave as thermally thin materials, such as a film or fiber. After ignition in stage (i), the very top layer of FPUFs at d_0_ is consumed under the influence of thermal radiation, forming a thin pyrolysis zone at the first pyrolysis temperature (T_p1_). The yielded liquid pyrolysis products mainly belong to the soft segments, and the volatiles released mainly belong to the hard segments. The excellent thermal insulation of the foam results in a rapid decrease in temperature across the intact foam, as the entire unmolten part is thermally thick. As heating continues, stage (ii) is reached, with the next few layers from the top of the FPUF also collapsing and melting, forming a thicker pyrolysis zone. Due to the good convection of the molten melt from d_1_ to d_2_, the melt is considered to reach the same pyrolysis temperature at T_p1_. As the remaining unburned material is still thermally thick, its temperature decreases inversely toward the bottom of the material. The cellular structure melts and collapses in stage (ii), resulting in a pool fire (iii), generally at the second pyrolysis temperature (T_p2_) from d_3_ to d. The high fluidity of the melt under high temperatures exhibits a constant temperature due to convection. Almost no heat flux is attributed to further heating in stage (iii), but the heat flux is completely transferred to pronounced pyrolysis. 

RPUFs behave quite differently from FPUFs during burning, showing the typical HRR curve for residue-forming materials in Figure 3 [9,26,27]. After ignition in stage (i), they reach the PHRR immediately, undergoing distinct charring at T_p1_, with no structural collapse and no formation of a pool fire because of their higher crosslink density. The char on the top acts as a protective layer, shielding the material underneath. The PHRR is subsequently followed by steady burning in stage (ii) at a lower HRR. The pyrolysis front at T_p2_ continuously consumes the material downward from the top (d_0_) to d_1_. Due to the effective protective layer formed, the temperature of the unburned material from d_1_ to d decreases inversely toward the bottom of the material. The length of the steady-burning phase in the HRR curve depends on the amount of combustible material [26]. Therefore, less heat is released by RPUFs as the char yield increases [9,27]. In stage (iii), the pyrolysis front at T_p2_ moves to the bottom of the material, and the flame is finally extinguished. 

### 2.2. Role of Selecting Contents of Isocyanate, Polyol, Foaming Agent, and Flame Retardants

**Figure 4:**
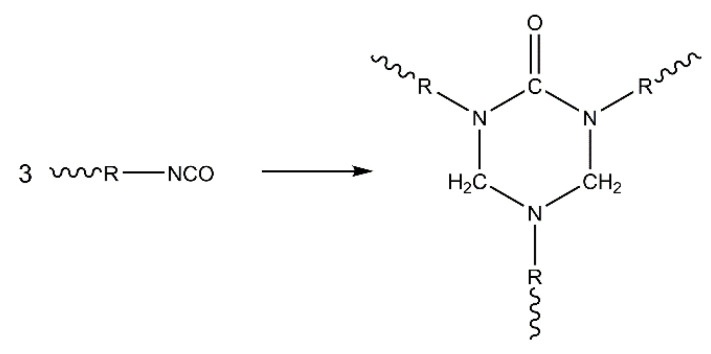
Trimerization of isocyanates.

Polyurethane chemistry is based on the high reactivity of isocyanates. Diisocyanates are organic compounds with two isocyanate groups, which are widely used to link polyols together through an exothermic reaction between isocyanates and hydroxyl groups in order to build crosslinked polyurethane. The content of diisocyanates in the formulation influences the thermal stability, rigidity, and fire behavior of PUFs. For instance, any excess isocyanates are converted into trimers by trimerization (see Figure 4), called isocyanurate rings [52]. Isocyanurates improve flame retardancy because the presence of a ring structure facilitates charring, forming a protective layer in the condensed phase. Apart from isocyanurates, side products such as polyurea with urea linkages are formed through the reaction of isocyanates with amine-terminated compounds. Polyurea provides the foam with rigidity and thermal stability.

**Figure 5:**
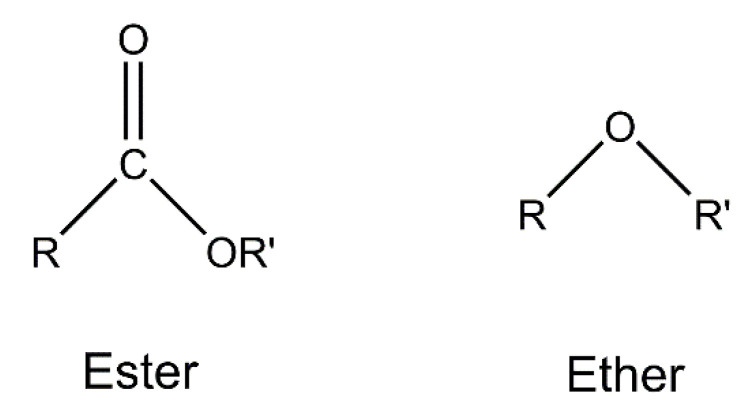
Ester and ether bonds.

Polyester polyols and polyether polyols are the two main types of polyols. The difference in chemical structure between ester and ether is shown in Figure 5. Polyether polyols have more resistance to hydrolysis but are less stable to oxidation; the inverse is true for polyester polyols. Polyurethane foams based on polyether polyols have a lower decomposition temperature in air than those based on polyester polyols. To improve the fire behavior of PUFs, flame-retardant polyols such as VORAGUARD ^TM^ Polyol from The Dow Chemical Company (Midland, MI, USA) and Exolit^®^ OP 560 from Clariant AG (Muttenz, Switzerland) are used. Another way to enhance the flame retardancy of PUFs is to use aromatic polyols to promote char yield during burning [53]. 

The blowing agent is a factor that is believed to influence the burning behavior of RPUFs due to their closed-cell structure. Chemical and physical blowing agents can be used to encourage the foaming process. Water acts as a chemical blowing agent that reacts directly with isocyanates to release carbon dioxide, which is an inert gas. Pentane, cyclopentane, and hydrofluorocarbon are common physical blowing agents [54]. Physical blowing agents are flammable, so they bring a degree of flammability to the closed-cell structure of RPUFs. Physical blowing agents are trapped in the foam and act as additional fuel during burning. Therefore, the selection of suitable blowing agents may also be significant for the flame retardancy of RPUFs.

Effective flame retardants help to improve the fire behavior of materials by increasing the time to ignition and decreasing the HRR to diminish fire spread. The selection of flame retardants usually depends on the structure–property relationship, processing, compatibility with the polymer matrix, costs, and the applications of polymeric materials [55]. However, there is no all-rounded flame retardant that can be applied to all materials. Adding char promoters such as phosphorous compounds is beneficial to the flame retardancy of RPUFs because the structure of rigid foams is favorable to char due to the high crosslink density during burning. In this case, a higher yield of char residue is generated, and the protective layer formed provides better thermal insulation to the material in the condensed phase, thus releasing less heat. A high loading of additive-type flame retardants is usually required for FPUFs to achieve the desired flame retardancy; however, it usually results in poor mechanical properties of the material. It is suggested that using reactive-type flame retardants is a good strategy to improve the fire behavior of FPUFs while limiting their influence on mechanical properties. Besides using a single flame retardant, combining two flame retardants or even more in one polymer system has made an excellent impression on researchers and the industry, as the right combination of flame retardants can create excellent flame retardancy.

### 2.3. Effective Flame-Retardant Approaches

The flame-retardant modes of action fall into two categories, namely, those that take place in the condensed phase and those that take place in the gas phase. The flame retardants that work in the condensed phase enhance carbonaceous char, reducing the release of combustible volatiles and acting as a protective layer to reduce the mass loss rate, and in some systems, to cause incomplete pyrolysis. Gas-phase flame retardants release non-combustible gases during decomposition to reduce the effective heat of combustion by fuel dilution or release radical scavengers to reduce the combustion efficiency (χ) of the flame (flame inhibition). Extremely active OH· and H· free radicals form during the burning of hydrocarbon fuels, and the system is subjected to an exothermic oxidative chain reaction [56]. To reduce the heat release from the reaction, reactive radicals are scavenged from the gas-phase flame retardants to replace OH· and H·. It is an efficient way to inhibit the flame, but smoke and CO yield are increased. Many outstanding flame retardants exhibit several mechanisms in parallel, such as flame inhibition and a melt-flow retreat effect [57]. Zammarano et al. [58] studied the heat release rate (HRR) and melt dripping of FPUFs with carbon nanofibers, and the results showed that the system successfully built an entangled fiber network that eliminated melt dripping by increasing the viscosity of the melt and thus formed a protective layer on the surface of the polymer matrix to reduce the HRR. Kempel et al. [59] analyzed the competitive and collaborative relationship among melt dripping, gasification, charring, flame inhibition, and combustion through the particle finite element method in order to understand the complex behaviors of polymeric materials during UL 94 testing. In conclusion, there are two combinations of flame-retardant approaches that serve as effective strategies to enhance the flame retardancy of foams: (1) flame inhibition + enhancement of melt flow and dripping; (2) charring + maintaining structural integrity of the foam or fire residue.

(1) Flame inhibition + enhancement of melt flow and dripping

Flame retardancy can be improved by the combination of flame inhibition in the gas phase and a retreat effect due to increased melt flow in the condensed phase [56,59]. The most important factor affecting the dripping behavior of polymers in fire is melt viscosity. A polymer with low melt viscosity tends to drip during combustion. Although melt flow and dripping can be detrimental to the burning polymers, at the same time, they offer an opportunity to slow flame spread or even cause extinguishment, as they remove mass and heat from the pyrolysis zone [60]. For instance, the flame inhibition of PUFs can be achieved by releasing compounds containing phosphorus during burning, and melt flow and dripping can be enhanced by plasticizers or radical generators in the condensed phase.

(2) Charring + maintaining structural integrity

Flame retardants produce carbonaceous char in the condensed phase that forms a layer that protects the material underneath. However, these char layers are usually fragile and easily form cracks or even collapse, resulting in the exposure of the underlying unburned material to the flame and causing some side burning. Therefore, maintaining the structural integrity of the foam or intumescent fire residues and the mechanical and thermal stability of char is a way to reinforce the barrier to the underlying material against heat and mass transfer.

## 3. Burning Behavior of Polyurethane Foams with a Single Flame Retardant

EG and phosphorus compounds are quite commonly proposed as effective single flame retardants in PUFs [61,62,63,64,65]. EG and phosphorus have their own specific flame-retardant modes of action and behave differently during burning. In this section, the details of EG and phosphorus compounds as flame retardants in PUFs are individually discussed.

### 3.1. Expandable Graphite

**Figure 6:**
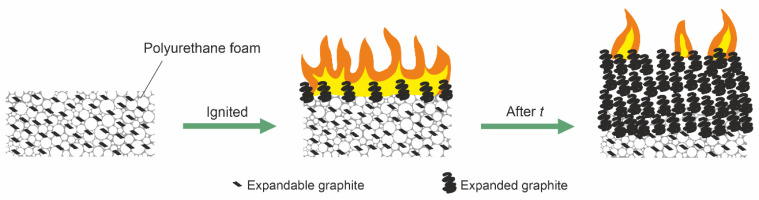
Burning behavior of PUFs with expandable graphite.

Natural graphite inherently has a layered structure. Intercalation is an important process to turn natural graphite flakes into EG. Therefore, EG is usually prepared by inserting oxidants, such as sulfuric acid, nitric acid, phosphoric acid, and acetic acid, between the layers of graphite [66]. The acid decomposes into gases, causing the graphite layers to be forced apart, thereby expanding graphite during heating. It mainly acts in the condensed phase by enhancing the char yield [63,65]. The burning behavior of PUFs with EG is illustrated in Figure 6. EG expands in size by several hundred times, developing a loose, porous “worm-like” structure to form a low-density thermal insulation layer, thereby protecting the underlying material from the heat source and slowing down pyrolysis by decreasing the release of volatile compounds. A minor factor in reducing flammability is that EG releases incombustible gases, such as CO_2_, SO_2_, and H_2_O, which helps to dilute the combustible gases surrounding the flame [38,66]. As the temperature rises, the sulfuric acid reacts with graphite, which leads to the oxidation of graphite to form CO_2_, water, and SO_2_, thus increasing the volume of EG to provide flame retardancy to the materials. 

C + 2H_2_SO_4_ → CO_2_ + 2H_2_O + 2SO_2_ [62].

**Figure 7:**
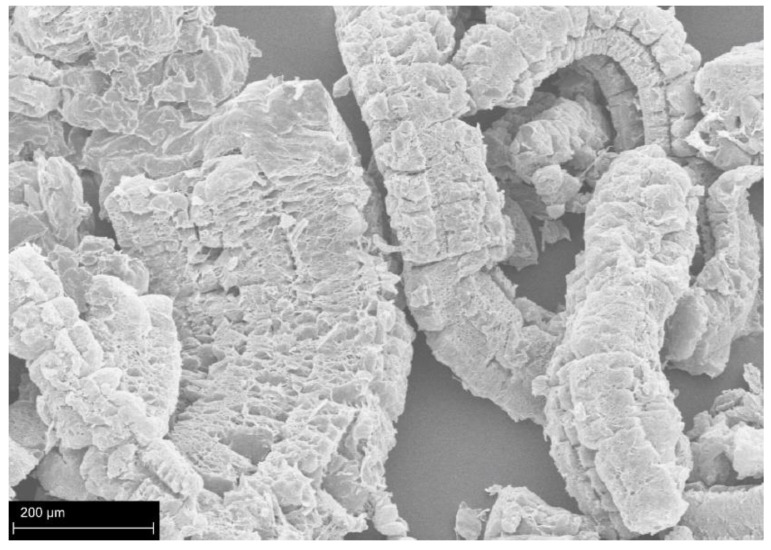
Scanning electron microscope (SEM) image of expanded graphite.

However, as shown in Figure 7, expanded graphite is usually fragile and loose. Due to the low adhesion of expanded-graphite char, cracks are easily formed, and more heat flux is exposed to the underlying polymer matrix. Improving the flame retardancy of PUFs by increasing the amount of EG is a challenge. Greater amounts of EG tend to deteriorate the mechanical properties, because EG acts as a nucleating agent to disrupt the structure of the foam [61,67]. In addition, the thermal insulating performance is diminished, and electrical conductivity is increased though the solid phase of conductivity of EG. 

**Figure 8:**
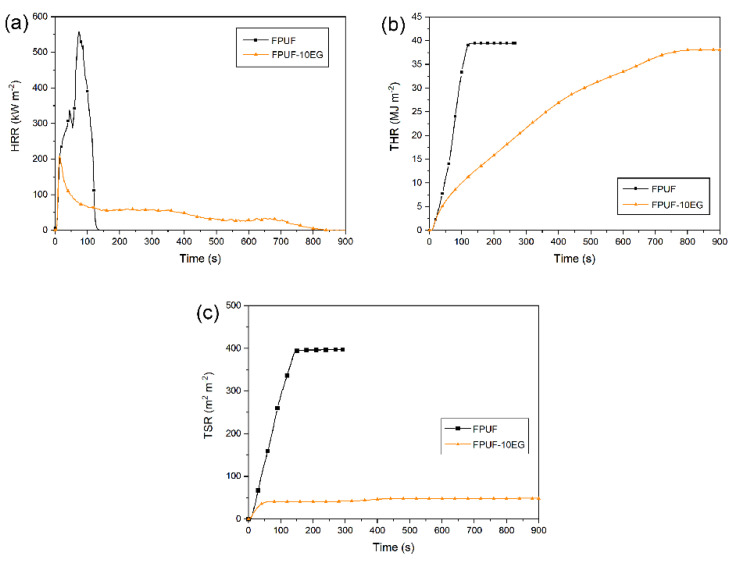
(**a**) Heat release rate (HRR), (**b**) total heat release (THR), and (**c**) total smoke release (TSR) of FPUFs and FPUF-10EG.

The burning processes of FPUFs and FPUFs with 10 wt.% EG (FPUF-10EG) are described by the heat release rate (HRR) and total heat release (THR) curves in Figure 8a,b, respectively, via cone calorimeter measurement. With 10 wt.% EG, the HRR is greatly reduced, and the sharp peak appears at the beginning of burning [42,68]. The very top surface of the polymer matrix is exposed to the heat flux, initially without any protection, so that the HRR reaches the highest value within a very short time. Since the FPUF with 10 wt.% EG in the pyrolysis front region is continuously subjected to the pyrolysis temperature, the polymer matrix starts to decompose. After accumulating a certain amount of expanded graphite at the pyrolysis front, it acts as a protective layer for the underlying material. The HRR keeps gradually decreasing, and the burning time is prolonged. The presence of 10 wt.% EG results in a lower PHRR and a flatter HRR curve. Only minor second and third peaks following the PHRR are shown in the HRR curve of FPUF-10EG, which proves that a sufficient amount of EG significantly reduces the fire hazard.

Apart from enhancing the flame retardancy of PU foams, EG performs through smoke suppression, as shown in Figure 8c [69]. The higher the amount of EG added is, the less smoke is released. EG reduces the smoke generated during the burning process because expanded graphite prolongs the residence time of smoke precursors in the pyrolysis zone, charring more aromatics, while expanded graphite protects the underlying materials, thus causing less polymer matrix to be consumed [70,71,72].

According to the UL 94 test, there are two common modes regarding dripping: (1) dripping with flame and (2) dripping without flame. The former may propagate the fire to the flammable materials nearby, enhancing the fire. The latter mode is achieved by removing the heat and fire load to cause dripping without any flame and prevent the propagation of the fire, diminishing the fire due to less heat and fewer flammable components in the burning material. However, a sufficient amount of EG uses a different mode from the above. It reduces the melt drips throughout the test to limit flame spread [61,73]. Because the intumescent structure of expanded graphite provides many tiny openings to keep the melt from dripping and because the char residue is not combustible, EG functions as an anti-dripping agent [73,74,75,76].

#### Flame-Retardant Performance Optimization of Expandable Graphite

The properties of EG, such as expansion volume, particle size, and type of intercalant, determine its effectiveness as a flame retardant in PUFs. Acuña et al. [61] showed that a higher expansion volume of EG improved flame retardancy and reduced smoke production because the larger particle size of expanded graphite provided a compact protective layer to reduce the heat flux passing through to the material underneath. They concluded that the particle size of EG is a key parameter affecting flame retardancy. Pang et al. [77] studied how the EG size affects the flame retardancy of rigid polyurethane foams with EG and ammonium polyphosphate (APP). The study shows that the size of EG had a linear relationship with the expandable volume. They proved that a greater size of EG provided greater flame retardancy, with a higher limiting oxygen index value and increased char yield. The addition of EG and APP delays the decomposition reaction and strengthens the char residue through the formation of a phosphorus-carbonaceous polyaromatic structure. Li et al. [78] confirmed that the larger particle size of EG was advantageous to the synergistic effect between EG and APP in semi-rigid polyurethane foams, as it formed a more continuous and compact protective layer that effectively shielded the transmission of heat to underlying materials during burning. 

Apart from particle size, the type of intercalants between the graphite layers is a decisive criterion for enhancing the flame retardancy of PUFs. Lorenzetti et al. [38] investigated the effect of the intercalants of EG on the flame retardancy of polyurethane foams. They observed that the PUF with sulfur-intercalated EG performed better than that with phosphorus-intercalated EG in terms of flame retardancy.

### 3.2. Phosphorous Flame Retardant

**Figure 9:**
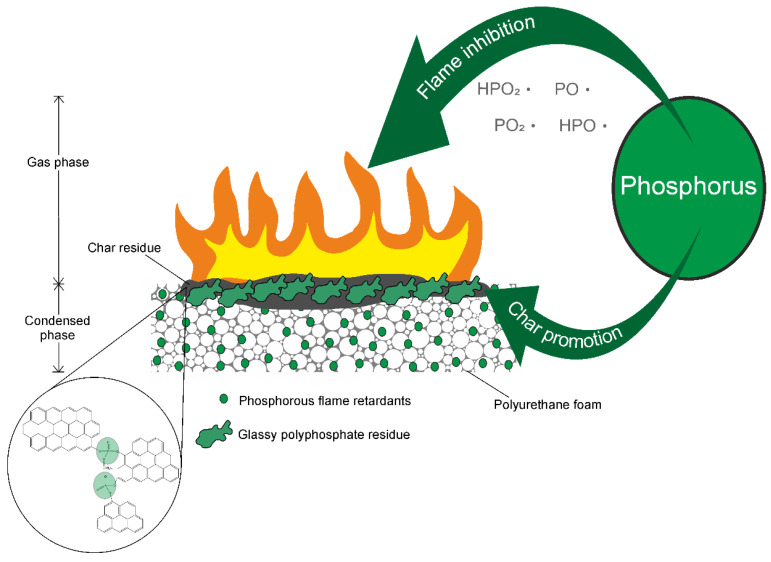
Modes of action of phosphorous flame retardants.

Figure 9 shows the modes of action of phosphorous flame retardants [56]. Phosphorus usually works in the gas phase and the condensed phase [79,80,81]. The free phosphorous radicals, such as HPO_2_·, HPO·, PO·, and PO_2_·, generated in the gas phase can quench the other free radicals formed, such as H· and OH·, by slowing down or interrupting the branching and chain reactions of the oxidation of hydrocarbons during burning, thus playing a role in flame inhibition [82,83]. Phosphorous radicals lead to less complete combustion in the flame zone, thereby reducing combustion efficiency (χ). As a result, increased amounts of incomplete combustion products such as smoke and carbon monoxide evolve at the same time [2,32]. Meanwhile, the heat release is reduced because phosphorus prevents the conversion from carbon monoxide to carbon dioxide, which is a highly exothermic reaction. In the condensed phase, phosphorus takes a variety of modes of action. The dehydration reaction of the polymeric structure during burning induces aromatization and graphitization, and phosphorus acts as a crosslinker to enhance charring. Bourbigot et al. [84] demonstrated that polyaromatic species are crosslinked with phosphohydrocarbonaceous bridges to form voluminous carbonaceous char with higher thermal stability. Phosphorus generally pyrolyzes under elevated temperatures, forming phosphoric acid derivates to catalyze the carbonization of polymers. However, some phosphoric acid, instead of interacting with the charring agent, generates inorganic polyphosphate glass that acts as a barrier to reduce mass transfer and heat release [85,86,87]. Although phosphorous compounds are char promoters, incomplete charring by phosphorus, such as aromatization without graphitization, can increase smoke release and even produce larger decomposition fragments. Phosphorous compounds are used as additive or reactive flame retardants in polyurethane foams. For the former, the flame retardancy of the material may decrease over time due to the migration of the flame retardant. Moreover, flame-retardant additives are usually detrimental to the mechanical properties of polymers. Conversely, reactive phosphorous flame retardants are chemically bonded to the main polymer chain or grafted to the backbone as branches. Therefore, using reactive flame retardants is a solution to prevent migration, providing even distribution on the polyurethane backbone and maintaining mechanical performance.

#### Flame-Retardant Performance Optimization of Phosphorus

Beyond decomposition and evaporation temperatures, the phosphorus oxidation state of phosphorous flame retardants determines their reaction rates with the carbon source and plays an important role in the flame-retardant efficiency of PUFs [4]. Phosphorous flame retardants with different phosphorus valence behave differently in various modes of action. Lorenzetti et al. concluded that the lowest phosphorus valence (+1) was active in both the gas and condensed phases, while the highest phosphorus valence (+5) only worked in the condensed phase [88]. Lenz et al. compared phosphorous flame retardants with different phosphorus oxidation states (+1, +3, +5) [89]. They observed that the phosphorous flame retardants with the lowest phosphorus valence (+1) were more effective in the gas phase. Chen et al. also reported that phosphorous flame retardants with the lowest phosphorus valence (+1) were likely to function in the gas phase and provided better flame retardancy than those with higher phosphorus valence [90]. The mode of action of phosphorous flame retardants during decomposition can be predicted, and the flame retardancy of PUFs can be optimized by choosing the phosphorous flame retardants according to their decomposition and phosphorus oxidation state. The concentration of P-FR used is also a key to optimize the flame-retardant performance of PUFs. With the increase in the concentrations of P-FRs used in the polymer, flame retardancy is significantly improved. Over a certain amount of P-FR concentration, flame retardancy tends to be stable or even decline [91,92]. Thus, flame retardancy is somewhat limited when a P-FR is used alone. Synergy in multicomponent systems is one way to improve the flame retardancy of polymers [93,94]. 

## 4. Mechanism of Synergistic Effect between Phosphorus and Expandable Graphite

The combination of phosphorus and EG is an advantageous approach to obtain efficient flame retardancy, and at the same time, the flame-retardant content can be kept as low as possible to reduce the worsening of the mechanical properties [95]. As both EG and phosphorous flame retardants have their own strengths in flame retardancy, they can complement each other. General synergy between phosphorus and EG occurs when flame inhibition and the protective layer are combined [43]. Combustion in the flame and pyrolysis can be understood as two strongly coupled chemical reactions [96]. In addition, at the beginning of burning, when the protective layer is still built up, flame inhibition can delay ignition and/or reduce the first pHRR [18,73,97]. 

**Figure 10:**
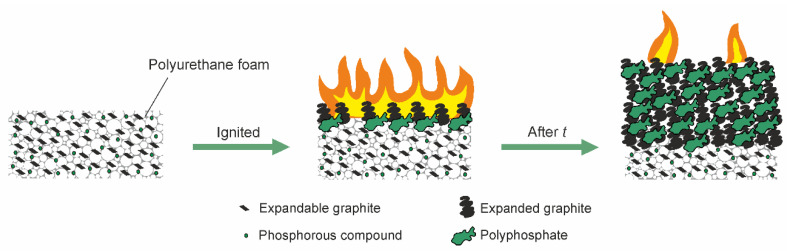
Burning behavior of PUF with expandable graphite and phosphorous compound.

**Figure 11:**
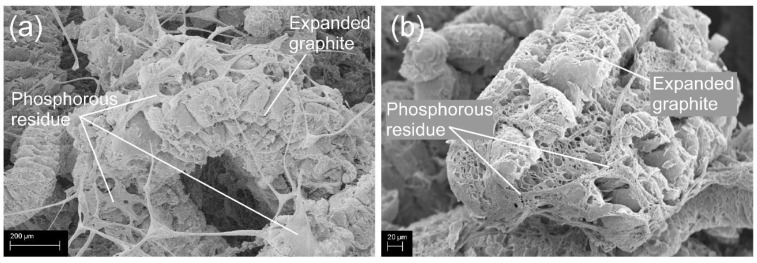
Phosphorous residue acts as a binder for expanded graphite in FPUFs.

**Figure 12:**
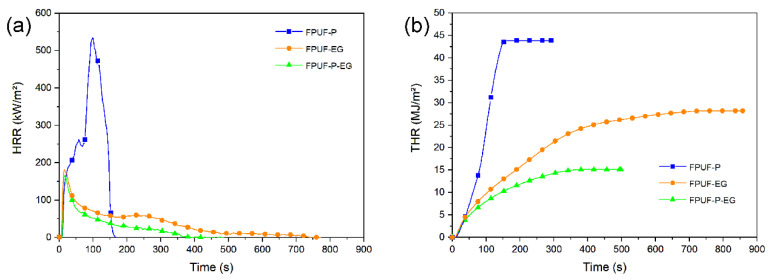
(**a**) Heat release rate and (**b**) total heat release of FPUF-P, FPUF-EG, and FPUF-P-EG.

Many contributions to the literature have stated that distinct synergistic effects occur between EG and phosphorus, especially regarding the weight and the morphology of char residue [98,99,100]. Any synergistic interaction between FRs active in the condensed phase is not straightforward [101] but only occurs when specific mechanisms enhance their efficiency [102]. Figure 10 depicts the burning behavior of PUFs with EG and a phosphorous compound. After ignition, the top layer of EG expands, and the phosphorous compound decomposes to form glassy polyphosphate. The cohesion of the fluffy expanded graphite increases because char is glued together by this polyphosphate. It strengthens the char structure and provides a superior protective layer against the external heat flux for the unburned underlying material [103,104,105]. Figure 11a,b are SEM images showing that expanded graphite is surrounded by the phosphorous residue, which strengthens the char layers. The phosphorous residue acts a binder to maintain the integrity of the carbonaceous char by linking the expanded-graphite particles. The adhesion of carbonaceous char effectively prevents the formation of cracks during burning to protect the underlying materials. Thus, the total heat release (THR) decreases crucially because of incomplete burning. Figure 12 displays the HRR and THR of FPUF samples with phosphorus (FPUF-P), EG (FPUF-EG), and phosphorus/EG (FPUF-P-EG). FPUF-EG and FPUF-P-EG significantly reduce the peak heat release rate (PHRR) and the THR when compared with FPUF-P. FPUF-P-EG shortens the burning time of FPUF-EG even further, because the combination of phosphorus and EG creates a better protective layer for the underlying material. The underlying material undergoes incomplete pyrolysis or even stops decomposing due to less heat transfer, thus simultaneously reducing the THR.

## 5. Current and Future Tasks

Due to the current general trend and upcoming environmental regulations, further breakthroughs are still ahead, both in the manufacture of PUFs and with regard to the flame retardants used in PUFs.

### 5.1. Green Solutions for Flame Retardants

Inventing various novel halogen-free chemical flame retardants, using solid waste-based fillers [106,107,108], and developing environmentally friendly flame-retardant additives in polymeric materials are the current trends in sustainable development. Surprisingly, flame retardants not only exist in laboratories but can also be found in nature. The use of natural flame retardants is an environmentally friendly approach. Some of the natural compounds can be used directly, while others require certain modifications before they can be used as flame retardants. 

#### 5.1.1. Natural Renewable Resources as Flame-Retardant Additives

**Figure 13:**
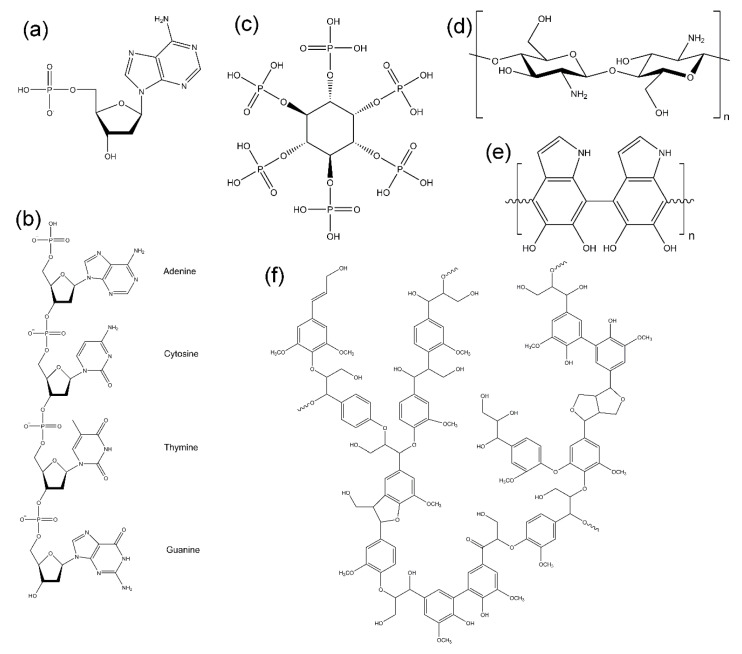
Natural flame-retardant additives: (**a**) deoxyribonucleic acid (DNA), (**b**) chain of DNA, (**c**) phytic acid, (**d**) chitosan, (**e**) polydopamine, and (**f**) lignin.

Some biological resources can be added to polymeric materials to enhance flame retardancy due to their special chemical structure and/or content of flame-retardant moieties. One of the natural flame retardants is deoxyribonucleic acid (DNA). DNA is responsible for the storage of genetic information about organisms. The chemical structure of DNA shown in Figure 13a exhibits a carbon backbone that connects with phosphate groups and nitrogen-rich nucleobases (adenine, guanine, cytosine, and thymine). As shown in Figure 13b, phosphodiester linkages form the backbone of DNA, linking nucleotides together. DNA can be used as an intumescent flame retardant because the three main constituents of DNA (phosphate, pentose, and nitrogenous base) are similar to the three chemical components of a traditional intumescent system: a char promoter, a char source, and a blowing agent [109]. During the combustion process, a foamed carbonaceous protective layer is formed, providing thermal insulation to limit the transfer of heat and fuel between the flame and the polymer. By studying the thermal decomposition process, Alongi et al. [110] found that the ceramic-like intumescent protection layer formed by DNA had higher thermal stability than the intumescent char formed by traditional intumescent flame retardants. Li et al. [111] used DNA-based nanocomposites as a bio-coating to increase the flame retardancy of FPUFs. 

Phytic acid is a bio-based flame retardant rich in phosphorus. The chemical structure is shown in Figure 13c. Relative to its molecular weight, it contains around 28 wt.% phosphorus. The phosphate group acts as a char promoter upon burning. Sykam et al. [112] reviewed different research papers, focusing on the flame retardancy of phytic acid applied to cotton and wool fabrics. Phytic acid catalyzes the carbonization of cellulose fibers to form a dense carbonaceous layer, which protects the heat transfer within the unburned material below the flame. Phytic acid not only works in the condensed phase, but also in the gas phase. When phytic acid combines with blowing agents such as ammonium ions and amine compounds, an expanded char foam is formed, providing stronger thermal insulation. Lin et al. [113] conducted research on layer-by-layer (LbL) coatings of Ti_3_C_2_, phytic acid, and chitosan for FPUFs. They found that the FPUF coated with Ti_3_C_2_/phytic acid/chitosan had better flame retardancy than that with only Ti_3_C_2_/chitosan. Compared with the FPUF coated with Ti_3_C_2_/chitosan, the peak HRR and total smoke release (TSR) of the FPUF coated with Ti_3_C_2_/phytic acid/ chitosan were reduced by 51.1% and 84.8%, respectively. The phosphorus in phytic acid acts as a char promoter in the polymer matrix to increase the char yield.

Chitosan, shown in Figure 13d, is a fibrous compound extracted from crustacean shells. Wong et al. [114] coated FPUFs with chitosan and EG using single-step coating. In the cone calorimeter measurements, the combination of chitosan and EG in FPUFs significantly reduced the PHRR, THR, and TSR. Compared with uncoated foam, the char yield of the FPUF containing chitosan and EG was increased by more than six times. Chitosan is also commonly used as a layer-by-layer (LbL) coating material. Nabipour et al. [115] coated FPUFs with nine bilayers of alginate, chitosan, and hydroxyapatite. The nine-bilayer-coated PUF showed reductions in PHRR and smoke production rate (SPR) of 77.7% and 53.8%, respectively. Lin et al. [116] coated FPUFs with eight bilayers of Ti_3_C_2_ and chitosan. The coated foam reduced the PHRR and TSR by 57.2% and 71.1%, respectively. Coating containing chitosan provides excellent flame retardancy for FPUFs.

Polydopamine (PDA), illustrated in Figure 13e, is a polymeric product yielded by the self-polymerization of dopamine, which is a hormone and neurotransmitter found in various organisms [117]. The advantage of using PDA as a coating material is that it has high adhesion to the surface of various materials. Cho et al. [118] conducted a study on the flame retardancy of PDA-coated FPUFs. They found that the PHRR of the PDA-containing FPUF with a PDA coating thickness of 240 nm (PDA coating for 72 h) was 67% lower in cone calorimeter measurements than that of the uncoated FPUF. PDA works in both the gas phase and condensed phase because it contains nitrogen and is composed of aromatic rings [119]. 

Lignin, displayed in Figure 13f, mainly provides structural support to plants and is found in cell walls. Lignin is used as a charring agent or flame retardant, because it produces high char yield during burning due to its high weight percentage of aromatic structure with respect to molecular weight [120,121]. Unmodified lignin was added directly to RPUFs and FPUFs [122,123]. Lignin is a polyol typically used as a filler in FPUFs to increase the viscosity of the pyrolysis products to prevent dripping [46]. However, attention must be paid to the amount of lignin added to PUFs, because there are functional hydroxyl groups on lignin that may react with isocyanates during the foaming process, thereby increasing the proportion of hard segments and causing the PUF structure to become brittle, affecting the mechanical performance.

It is noteworthy that pure lignin is composed of functional hydroxyl groups that can be modified to improve flame retardancy and compatibility with polymer matrices [124]. Phosphorylated lignin is a typical example of combining phosphorus and a charring agent in one flame retardant [8]. Xing et al. modified lignin with phosphorus for RPUFs, replacing the petroleum polyol with phosphorylated lignin [125]. Their research study proved that the combination of modified lignin and phenolic encapsulated ammonium polyphosphate in RPUFs reduced the HRR and THR and increased the char yield. Zhang et al. [126] synthesized 9,10-dihydro-9-oxa-10-phosphaphenanthrene-10-oxide-based lignin to improve the flame retardancy of polyurethane. They demonstrated that the formation of expanded carbonaceous char in the condensed phase by the DOPO-based flame retardant was indicative of an improved flame retardancy of polyurethane. More examples on the modification of renewable resources into functional flame retardants are discussed in Section 5.1.2.

#### 5.1.2. Modification of Renewable Resources into Functional Flame Retardants

Material scientists are seeking different bio-based flame-retardant solutions. Due to the excessive exploitation of fossil fuels, the use of renewable resources is a hot topic at present. More and more countries are aware of this problem and are introducing regulations to deal with the excessive use of non-renewable resources. It is environmentally friendly to modify bio-based resources into functional flame retardants in polymeric materials to improve flame retardancy and lighten the burden on the Earth. 

The most common method for preparing bio-based flame retardants is to take advantage of the chemical similarity between petroleum polyols and plant oils. Plant oils are used in PUF formulations to increase the bio-content of the material from an environmental perspective. Most plant oils, for example, soybean oil [127,128], palm oil [129], and linseed oil [130], are composed of fatty acids that can be directly chemically modified into polyols by introducing hydroxyl groups at the position of the double bonds. The functional groups can be modified by hydroformylation, hydrolysis, ozonolysis, and epoxidation [131]. Among them, epoxidation is a common way to modify functional groups. Fatty acids in plant oils are mostly unsaturated and are reactive to form epoxy rings through epoxidation [132]. The hydroxyl group can then be formed by opening the epoxy ring on the epoxidized oil [133,134]. Therefore, plant oils may react with isocyanates to form urethane bonds. However, plant-oil-based polyols are usually more flammable than their petrochemical counterparts, because the hydroxyl groups formed are usually located in the middle of the fatty acid, with the remaining fatty acid chain treated as a dangling chain [135]. These dangling aliphatic chains serve as a fuel source to support combustion. Considering ways to improve the flame retardancy of PUFs by using plant oil, many scientists have introduced flame-retardant elements, such as phosphorus and nitrogen, onto the backbone of plant oil to obtain flame-retardant polyols, thereby effectively preventing the migration of flame-retardant moieties. Tang et al. [136] synthesized phosphorous soybean-oil-derived polyols for RPUFs. Compared with neat RPUF, the RPUF with 12.3 wt.% synthesized polyols significantly reduced the PHRR, THR, and total smoke production (TSP) by 40%, 35%, and 49%, respectively. The charring performance of RPUFs was improved by introducing the synthesized polyol, and the carbonaceous residue acted as a stronger thermal barrier. Some studies have also combined the advantages of EG and phosphorylated plant oil to simultaneously improve the flame retardancy and bio-based contents of polyurethane foams [137]. In another study of ours [97], petrochemical polyols were partially replaced with novel phosphorus-grafted soybean-oil-based polyols in the formulation of FPUFs with additional EG. The results showed that the synergistic effect between phosphorus and EG increased the char yield by three times and effectively reduced the HRR and THR. Acuña et al. [138] modified castor oil with nitrogen and phosphorous compounds into flame-retardant polyols combined with EG and graphene oxide (GO), which provided superior flame retardancy for RPUFs. Zhang et al. [139] synthesized phosphorous bio-based polyols using castor oil and diethyl phosphate as raw materials. EG was blended into the RPUF formulation. The result showed that the system with EG and phosphorus-grafted castor oil exhibited a large reduction in PHRR compared with the one with EG and glycerolysis castor oil. Chen et al. [140] fully substituted the petroleum-derived polyols in polyisocyanurate foams with phosphorous soy-based polyols they synthesized themselves, also adding EG and a commercial phosphorous liquid flame retardant. Flame retardancy was strongly enhanced by the combination of gas-phase and condensed-phase actions.

### 5.2. Green Solutions for Polyurethane Foams

Conventional polyurethane foams are mainly produced from petrochemical ingredients, polyols and diisocyanates. Due to increasing concerns about environmental protection, there is great demand for environmentally friendly products. Isocyanates, especially, cause environmental hazards and are highly toxic to human health. Thanks to scientific research regarding green solutions for PUFs, sustainable alternatives to polyols and isocyanates have been found, as well as different reactions to obtain urethane bonds.

CO_2_ has always been regarded as the chief culprit of global warming. The Covestro chemical company has capitalized on this waste. They have been researching and successfully producing CO_2_-based polyols for polyurethane via catalytic copolymerization [141]. They prepared FPUF from a 3-functional polyethercarbonate polyol and toluene diisocyanate. The apparent density, morphology, mechanical properties, and thermal stability of the CO_2_-based FPUF were comparable to those of the conventional variety. A starch unit was used to construct the structure of the soft segment by Lubczak et al. [142].

Most isocyanates on the market are derived from petroleum. To cope with the problem of non-renewable resources, bio-based alternatives to isocyanates have become available for PU. Konieczny et al. [143] reported that ethyl ester L-lysine diisocyanate and ethyl ester L-lysine triisocyanate were used to produce PU films. Hojabri et al. [144] synthesized fatty acid-derived diisocyanate to replace the petrochemical one for PU.

Conventional PU manufacturing processes use isocyanates, which are highly toxic to living organisms and unsustainable. In addition to the highly toxic isocyanates themselves, colorless toxic gas phosgene is used as a raw material in the manufacturing process of isocyanates [145]. Due to the health and environmental concerns about isocyanates, the synthesis of non-isocyanate polyurethane is a way to eliminate highly toxic compounds from the manufacturing process and final products. Non-isocyanate polyurethane can be synthesized though several reactions, polyadditon, rearrangement, polycondensation, and ring opening. The most general approach is cyclic carbonate–primary amine addition reaction [146,147]. During the formation of every urethane linkage, a primary or secondary hydroxyl group is also formed. This reaction yields polyhydroxyurethanes [148]. The reaction does not require the use of isocyanates. Cyclic carbonate can be directly synthesized by the reaction between the unsaturated bond and hydrogen peroxide to form an epoxy ring and subsequently react with carbon dioxide. However, certain carbonate–amine systems are less reactive, except at elevated temperatures and/or in the presence of a catalyst.

## 6. Challenges and Conclusions

The recyclability of PUFs is an important issue that needs to be addressed. Pure PUFs can be recycled and recovered through mechanical, physical, chemical, and thermo-mechanical processes. The main challenge is that PUFs containing traditional flame retardants cannot easily be recovered via pyrolysis. Flame retardants in PUFs change the decomposition temperature and may hinder the thermal decomposition of the material by charring [149]. Therefore, incineration is a common disposal method for flame-retardant PUFs. However, incineration has adverse effects on global warming due to the high emission of greenhouse gases. The recyclability of PUFs containing traditional flame retardants remains a challenge in practice. 

Biodegradation is an eco-friendly way to break down polymers. However, the bacterial degradation of PUFs takes an exceedingly long time because it largely depends on the structure and crosslink density of the materials [150]. The use of renewable resources continues to expand due to growing interest from the industry and academia. The trend is towards a safer, non-toxic, sustainable, and economical way to produce PUFs. Flame-retardant PUFs composed of fully sustainable ingredients, along with sustainable production methods, would also improve biodegradability as a solution for natural decomposition in the environment.

In addition to focusing on the environmental impact of the end-of-life disposal of PUFs themselves, the potential hazards of phosphorus-based flame retardants used in PUFs are also noteworthy. Since many small-molecule phosphorous flame retardants are not chemically bonded to the polymeric products, they can be released into the living environment through volatilization, leaching, and/or abrasion over time, and people can easily be exposed to them [151]. The potential health concerns phosphorous flame retardants present for human beings are considerable. Numerous studies have been conducted on the toxicity of phosphorus flame retardants for human health. Araki et al. investigated the impact of phosphorous flame retardants in residential dust on human health and reported that that their level was positively correlated with the prevalence of asthma and allergies [152]. Bruchajzer et al. found that phosphorous compounds affect reproduction in humans [153]. Nevertheless, phosphorous flame retardants have relatively low environmental toxicity compared with their halogenated counterparts [154,155]. In order to reduce the health hazards of phosphorous flame retardants for human beings, it is suggested to use reactive phosphorous flame retardants that are chemically bonded to the final products to avoid the leakage of phosphorus in the environment. In an environmentally friendly way, using the natural FRs mentioned in Section 5.1 instead of synthetic ones solves the problem of the chemical contamination of the environment. 

In addition to environmental and health concerns, meeting fire safety regulations is a major challenge in practical applications. The development of flame-retarded PUFs, especially FPUFs used in railway vehicles, which must fulfill the high requirement of a maximum average of the rate of heat emission (MARHE) value below 90 kW m^−2^ or even lower (based on the various sets of requirements) according to European standard EN 45545 “Fire Protection on Railway Vehicles”, is a particular challenge. 

Although the above-mentioned challenges question the application scope of PUFs, both academia and the industry are actively addressing them with successful and promising efforts. In terms of fire safety regulations for PUFs, the synergistic effects between phosphorus and EG provide impressive flame retardancy to PUFs and achieve a perfect balance between the mechanical properties and flame retardancy of PUFs. This feature article describes the fundamental mechanism of the synergistic effect between P-FRs and EG in PUFs. In further development, this synergy can create higher flame retardancy for PUFs with the right kinds and appropriate amounts of P-FRs and EG. In addition to the combination of P-FRs and EG, adding other flame-retardant elements, as well as cleverly adjusting the PUF chemistry to this combination, may be a further solution to provide PUFs with unexpectedly high flame retardancy through complicated interactions in the gas phase and the condensed phase [156,157,158]. The synergistic halogen-free combination of EG and P-FRs is posed as one of the current champions in the flame retardancy of PUFs and also offers the potential for a sustainable solution in future PUFs based on renewable polyurethane or with renewable flame retardants.

## Data Availability

Not applicable.
